# Patient Experiences of a Postpartum Cardiovascular Disease Intervention Clinic for Pregnancy Complications

**DOI:** 10.1007/s10995-025-04047-0

**Published:** 2025-02-07

**Authors:** Tegan Manthorpe, Margaret Arstall, Prabha H. Andraweera, Emily Aldridge

**Affiliations:** 1 Adelaide Medical School, Robinson Research Institute, The University of Adelaide, Adelaide, South Australia Australia; 2 Department of Cardiology, Lyell McEwin Hospital, Adelaide, South Australia Australia

**Keywords:** Postpartum intervention clinic, Pregnancy complications, Postpartum cardiovascular disease prevention, Cardiovascular disease in women

## Abstract

**Objectives:**

Experiencing a maternal complication of pregnancy conveys a significantly higher risk of developing premature cardiovascular disease compared to having an uncomplicated pregnancy. Postpartum interventions that aim to improve lifestyle and modifiable risk factors for people in this cohort may reduce cardiovascular disease risk. This study will explore the experiences and barriers to attendance of patients referred to one such clinic located in South Australia.

**Methods:**

This qualitative study conducted six focus groups comprised of two-six patients who had attended at least one postpartum intervention clinic appointment (*N* = 19). Audio recordings were captured and transcribed and NVivo was used to perform a thematic analysis.

**Results:**

Participants found the clinic informative as it educated them on their greater risk of cardiovascular disease and how to reduce this risk. They reported wanting more frequent appointments and the ability to opt in for additional contact, including newsletters and social media groups. We also identified several barriers to attendance, including an unclear clinic referral and appointment booking process, and missing clinic correspondence including appointment letters and pathology forms.

**Conclusions for Practice:**

This study provides insight into the experiences of patients who attended a postpartum cardiovascular disease prevention clinic. The clinic model can be operated in different health care settings to become part of standardized care in the postpartum period for patients who have had a pregnancy complication. Refinement of the clinic model referral and booking processes could reduce potential barriers to patient attendance.

## Significance

What is Already Known on this Subject?

What this Study Adds?

Previously, data from an international postpartum cardiovascular disease prevention clinic for patients who had a pregnancy complication reported that lack of time and inconvenience were barriers for engagement. No studies have investigated the perspectives of the attendees of the only existing, standard-care, postpartum, outpatient cardiovascular prevention clinic in Australia.

This is the first insight into the experiences of patients attending an Australian postpartum cardiovascular disease prevention clinic for patients who have had a pregnancy complication. Participants found the clinic appointments to be educational, practical and supportive, and wanted more regular appointments. The unclear clinic referral process and poor clinic correspondence were reported barriers and could contribute to the low attendance. This provides a clear indication of improvements that need to be made to the clinic to assist in maximizing patient outcomes.

## Introduction

### Cardiovascular Disease in Women

Cardiovascular disease, defined as conditions that affect the heart and blood vessels, is the leading cause of global morbidity and death (Australian Institute of Health and Welfare, 2019). In Australia, cardiovascular disease was the cause of > 30% of deaths in women in 2018 (Australian Institute of Health and Welfare, 2019). Despite this, the awareness of the high morbidity and mortality caused by cardiovascular disease in women is low (Dassanayake et al., 2020). The cardiovascular disease incidence and mortality rates for older age groups (> 55 years) and men has fallen in recent decades; however, these statistics have remained stable for younger women (< 55 years) (Wilmot et al., 2015). There are also disparities in care, with women experiencing greater treatment delays, less adherence to treatment guidelines and poorer outcomes compared to men (Adigun et al., 2018; D’Onofrio et al., 2015; Stehli et al., 2019). Women of low socioeconomic status and racial minorities also experience the worst outcomes, with less adherence to clinical treatment guidelines and higher mortality rates (Fabreau et al., 2014; Tavella et al., 2016). In addition, there are risk factors specific to women that generally do not feature in cardiovascular disease risk calculators and counselling, such as maternal complications of pregnancy (Arnott et al., 2020; Parikh et al., 2021).

### Maternal Complications of Pregnancy and Cardiovascular Disease

Pregnancy is considered a ‘cardiovascular stress test’, due to the significant maternal vascular, metabolic and physiological changes required to support fetal growth and development (Dassanayake et al., 2020; Parikh et al., 2021; Rich-Edwards et al., 2014). Inadequate adaptation to the physiological demands of pregnancy can result in the development of maternal complications of pregnancy, which are associated with an increased susceptibility to developing premature cardiovascular disease (Dassanayake et al., 2020; Rich-Edwards et al., 2014). The link between pregnancy complications and cardiovascular disease is still unclear (Aldridge et al., 2023). It is possible that pregnancy complications reveal those with underlying subclinical risks for cardiovascular disease or/and the pregnancy results in blood vessel changes thereby increasing risk (Dassanayake et al., 2020).

In Australia, approximately 30% of all pregnancies are affected by at least one maternal complication (Arnott et al., 2020). These include hypertensive disorders of pregnancy, gestational diabetes mellitus, spontaneous preterm birth, birth of a growth-restricted or small-for-gestational-age infant or placental abruption (Arnott et al., 2020). Patients with a history of at least one of these complications are at greater risk of premature cardiovascular disease than those who that have not had a complicated pregnancy (Grandi et al., 2019; Kramer et al., 2019; Parikh et al., 2021; Wu et al., 2018). The recurrence of some complications (preeclampsia and preterm birth) in multiparous women has found to be associated with a greater risk of cardiovascular disease than multiparous women who have had complications in only one pregnancy (Brouwers et al., 2018; Vounzoulaki et al., 2020).

## Interventions

Women with a history of a complicated pregnancy require early cardiovascular screening, education and intervention in the early postpartum period and beyond (Brown & Smith, 2020; Lewey et al., 2024). There are some broad clinical practice guidelines to manage future risk of cardiovascular for patients that have had a pregnancy complication (Brown & Smith, 2020; Whelton et al., 2018). The application of these guidelines to clinical practice however is inconsistent and there remains no consensus on the ideal time to commence cardiovascular education, screening or management for this large number of patients (Lewey et al., 2024).

### Engagement in Health Services

Engagement in preventative health services is crucial to improve clinical outcomes (Ellis et al., 2017). For instance, attending cardiac rehabilitation is associated with better cardiovascular outcomes, including lower rates of acute cardiac events and cardiac-related mortality, compared to not attending cardiac rehabilitation (Hamilton et al., 2018). Women with lower socioeconomic status engage less in preventative health services due to additional reported barriers of cost, lack of insurance coverage and distance to services (Arpey et al., 2017; Ellis et al., 2017). Studies into cardiac rehabilitation engagement has also found that women attend less than men, and consumers with lower socioeconomic status and high family responsibilities, or who require an interpreter are also less likely to attend (Supervía et al., 2017). Therefore, women, especially those living in low socioeconomic circumstances, need additional targeting for engagement with preventative health services.

### Current Study

Pregnancy complications are clearly related to cardiovascular disease; however, there are very few interventions (such as outpatient clinics) that aim to reduce this risk (Aldridge et al., 2020). Only one study has reported barriers to attending a cardiovascular risk prevention clinic for postpartum women after six months who have experienced obstetrical complications (Chan et al., 2021). Perceived barriers to engagement in the service were a lack of time and inconvenience in attending appointments; however, responses were limited by the use of short questionnaires (Chan et al., 2021). Additionally, no studies have investigated the experiences of consumers attending existing cardiovascular disease prevention clinics for those who experienced a pregnancy complication within Australia.

The postpartum intervention clinic at the Lyell McEwin Hospital in South Australia has been operating since September 2018 (Aldridge et al., 2020). There have however been no attempts to collect formal consumer feedback despite low patient attendance (Aldridge et al., 2023). It is unknown what this group of patients find beneficial, what the barriers to attendance are, or what could be improved to support the delivery and education. In addition, over two-thirds of clinic attendees were unaware of the higher risk of cardiovascular disease after pregnancy complications (Aldridge et al., 2023).

We conducted focus group discussions with patients who had attended the postpartum intervention clinic to explore their experiences and perspectives. The key topics for discussion were patient experiences of the clinic and areas of improvement to support attendance, engagement and education in the clinic.

## Methods

### Study Design

Focus groups were scheduled at a time when two researchers could be present, and a semi-structured interview guide was created to facilitate discussions. All eligible 128 participants were informed and invited to participate in the study. Focus groups were audio-recorded and transcribed. Ethics approval was received from The Central Adelaide Local Health Network Human Research Ethics Committee (Reference Number: 14436).

### Participants

Participants from this study were recruited from a postpartum intervention clinic at the Lyell McEwin Hospital in South Australia. The model of care for this clinic has been previously described (Aldridge et al., 2020). Briefly, to be eligible for referral to the clinic, patients must have experienced: a hypertensive disorder of pregnancy requiring medical therapy or resulting in delivery at < 37 weeks’ gestation; gestational diabetes mellitus requiring metformin or insulin therapy; spontaneous preterm labour (< 34 weeks’ gestation); intrauterine growth restriction or delivery of a small-for-gestational-age infant (< 5th customised birth centile); or placental abruption (Aldridge et al., 2020).

Appointments are scheduled at 6 months, 18 months and 5 years postpartum. At each appointment, data is abstracted from a combination of self-reporting tools and medical records by the clinic medical scientist. Anthopometrics, blood pressure and cardiometabolic blood and urine tests are also collected. The clinic nurse practitioner uses the collected information to provide indivualised health and lifestyle education.

Participants were eligible for the current study if they had attended at least one postpartum intervention clinic appointment and spoke English. Participants requiring interpreters were not eligible for inclusion in this study due to funding constraints.

### Focus Group Guiding Questions

1. What was your experience of having a pregnancy complication?
2. Was it clear why you were referred to the postpartum intervention clinic?
3. How did you find out about the postpartum intervention clinic?
4. What did you like?
5. Was there anything that could be improved, or you did not like?
6. How did you find the resources?
7. What do you think about the schedule of review appointments?
8. Would you like additional contact between postpartum intervention clinic appointments?
9. Any additional comments or things you would like to talk about/us to know?

### Statistical Analyses

Demographic information of the focus group study cohort was compared against the whole postpartum intervention clinic cohort. Demographic data were presented as counts and percentages for categorical variables or mean and standard deviation for continuous variables. Independent samples student t-tests were conducted to compare differences in means of continuous variables, and Fisher’s Exact test or Pearson’s chi-square tests were conducted to compare differences in proportions for two- and multi-category categorical variables, respectively. A p-value of < 0.05 was considered statistically significant.

The transcribed focus group interviews were analyzed with systematic text condensation (a four-step, cross-case method of thematic analysis commonly used for focus groups) using NVivo QSR International (Malterud, 2012). First, the focus group interviews were read to identify preliminary themes, then the preliminary themes were organized into code groups and subgroups and these codes were assigned meanings. Finally, the meanings were synthesized to descriptions.

## Results

### Participant Demographics

All eligible patients (*N* = 178) who had attended at least one postpartum intervention clinic appointment and did not require an interpreter were contacted regarding potential participation. Those who expressed interest were offered an appointment (*N* = 28). Nine patients who booked in were unable to attend on the day and could not be rescheduled for a later available focus group date. Attendees were offered a gift voucher to reimburse them for their time and encouraged to bring their children to ensure attendance. The final sample size included 19 participants, all of whom identified as women. All participants gave written informed consent prior to participating and were reminded of their right to withdraw at any time. Participant demographics compared with the complete postpartum intervention clinic cohort are included in Table 1.

**Table 1:** Participant demographics of a study into patient experiences of a postpartum cardiovascular disease intervention clinic for pregnancy complications

	Participant Demographics Mean ± Standard Deviation or *N* (%)		
	Focus Group Cohort *N* = 19	Overall Postpartum Intervention Clinic Cohort *N* = 261^#^	*p*-value
Age (years)	36 ± 4	33 ± 5	0.02*
Country of Birth Australia Other	12 (63) 7 (37)	143 (55) 118 (45)	0.30
Ethnicity Caucasian African subcontinent Southeast or Far East Asia Aboriginal Indian subcontinent Other	13 (68) 2 (11) 2 (11) 1 (5) 1 (5) 0 (0)	143 (55) 14 (5) 48 (18) 6 (2) 21 (8) 29 (11)	0.13
Indigenous Status Indigenous Non-Indigenous	1 (5) 18 (95)	6 (2) 255 (98)	0.77
Educational Level Lower than high school year 12 High school year 12 Technical and further education certificate/diploma University bachelor University postgraduate Unknown	1 (5) 2 (11) 6 (32) 12 (63) 2 (11) 2 (11)	31 (12) 35 (13) 101 (39) 50 (19) 16 (6) 28 (11)	0.30
Marital Status Married De-Facto Single	10 (53) 6 (32) 3 (16)	179 (69) 55 (21) 20 (8)	0.46
Occupational Status Employed Employed on maternity leave Unemployed	7 (37) 8 (42) 4 (21)	61 (24) 76 (30) 124 (48)	0.06
Combined Household Income <$20,000 $20,001 - $40,000 $40,001 - $70,000 $70,001 - $105,000 $105,001 - $205,000 Unknown/declined to answer	1 (5) 1 (5) 3 (16) 7 (37) 4 (21) 3 (16)	9 (3) 16 (6) 48 (18) 55 (21) 42 (16) 89 (34)	0.52
Pregnancy Characteristics Nulliparous Pregnancy Complication Hypertensive Disorder Gestational Diabetes Preterm Birth Placental Abruption Small for Gestational Age	6 (31) 6 (31) 15 (79) 1 (5) 0 (0) 1 (5)	101 (39) 87 (33) 187 (72) 5 (2) 4 (2) 17 (7)	0.63 0.54 0.33 0.32 0.74 0.64

* Statistically significant

^#^ Full cohort data presented, including from those who were not contacted for recruitment into focus groups

### Themes

Two major themes and related subthemes were identified when discussing the experiences of postpartum patients attending the postpartum intervention clinic. Another two themes and related subthemes were identified when discussing barriers to postpartum intervention clinic (Table 2). Saturation (the point at which coding consecutive focus groups reveal no new themes or subthemes) was reached after the third focus group (Hennink et al., 2019).

**Table 2:** Themes and subthemes of reported patient experiences and barriers in a postpartum intervention clinic

Aim	Themes	Subthemes
Patient Experiences	1. Patients found the clinic useful	1. Informative 2. Thorough in assessment 3. Practitioner gave practical recommendations 4. Provided motivation to make changes to reduce cardiovascular risk 5. Used resources provided 6. Made lifestyle changes to reduce cardiovascular risk
	2. Patients want more regular clinic appointments	1. Regular clinic appointments are important 2. Time between appointments is too long 3. Want yearly appointments 4. Want ability to book additional appointments 5. Want contact between appointments (phone calls, newsletters, online groups)
Barriers	3. The process of the referral and appointments was unclear	1. Not told about clinic referral during care 2. Received appointment letter and did not understand
	4. Missing and incorrect appointment letters and forms	1. Missing appointment letters 2. Did not receive pathology request forms 3. Received incorrect waiting list letter

### Aim 1. Reported Patient Experiences

#### Theme 1. Patients Found the Postpartum Intervention Clinic Useful

**Fig. 1:**
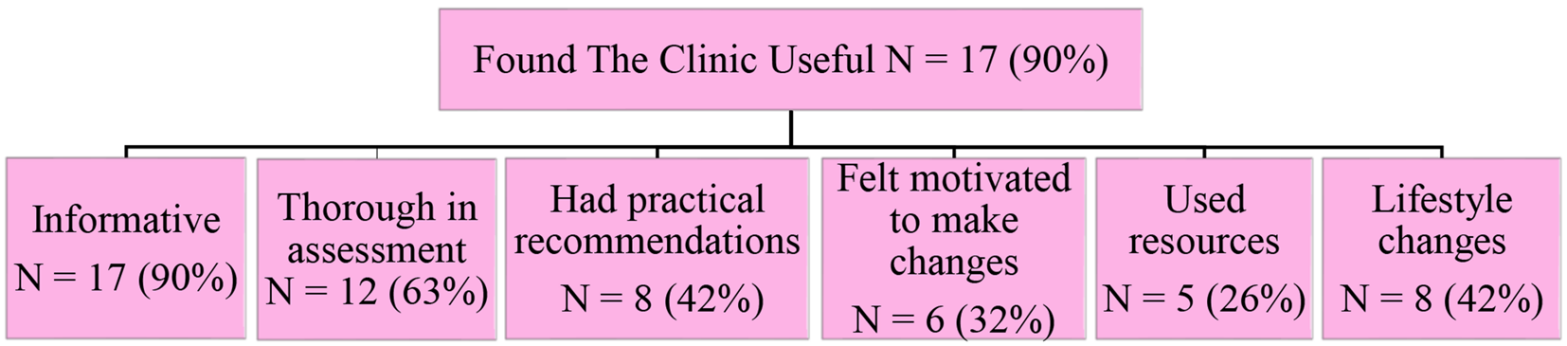
Patients found the postpartum intervention clinic useful and identified six subthemes in this area

The first emergent theme was that most attending patients (*N* = 17, 90%) found the postpartum intervention clinic useful (Fig. 1). Most (*N* = 17, 90%) found the clinic was informative in educating them about their risk of cardiovascular disease after having a pregnancy complication. Most participants (*N* = 7, 90%) reported that they were not previously aware of the link and only were made aware at the appointment:

Learning that this could have long term effects in the clinic beyond like potential heart disease, it was a shock, but I’d rather know. Nobody in hospital when I had preeclampsia said anything about it being a problem after birth. My doctor didn’t talk to me about it and none of my friends seem to know anything about it (participant 6).

Some participants also highlighted that the information provided on pregnancy complications and premature heart disease in the clinic was a stark contrast to the little information that they had received while pregnant after diagnosis with a pregnancy complication (*N* = 8, 42%):

The postpartum intervention clinic had the time to give me information and talk to me. It was so different to what happened when I got preeclampsia in my pregnancy. The doctors and midwives did not have a proper discussion with me about it. I did not even realize that it was a pregnancy complication until getting the letter about the postpartum intervention clinic (participant 7).

Most (*N* = 12, 63%) also described that the clinic provided them with information on how to reduce their risk of cardiovascular disease: “Now I know I need to keep my weight down and go for walks for my heart” (participant 14).

Patients also described the clinic assessments as thorough (*N* = 12, 63%): “It was everything, a proper check. We’re going to check your weight, talk about your health, talk about your food, how much exercise you get, and we’re going to talk about what you can do better” (participant 2). Some participants (*N* = 5, 26%) also talked about the thoroughness of the assessments by comparing it to appointments with other doctors or nurses who “only do one or two tests for what you have come to the appointment for that day. They always need to get you out the door quickly” (participant 19).

Eight participants (42%) also described some of the assessments as more comprehensive than previous screening tests they had before: “I haven’t had such a complete blood screen ever, even while pregnant. The phlebotomist who took my blood was stunned at how much she had to take from me, but it was good to check everything” (participant 10).

Almost half of participants (*N* = 8, 42%) also found that the nurse practitioner gave them recommendations that were practical to implement: “[the nurse practitioner] said try walking with the kids to school instead of taking the car and now we walk all the time together” (participant 13).

Six participants (32%) also found that the clinic gave them motivation to look after their cardiovascular health: “It was a reminder to look after myself. A kick in the shins to look after myself and do better. To not eat that or go for a walk with the pram” (participant 3).

Additionally, 5 participants (26%) described using the resources provided in clinic: “I got one of the guidelines and it’s on my fridge near the highchair, and I mentally try to check off the things I’ve eaten and haven’t eaten everyday” (participant 12).

Almost half of the participants (*N* = 8, 42%) also mentioned that they had made and maintained lifestyle changes after attending:

[The nurse practitioner] said the easiest thing I could do would be to cut out the sugar in my coffees, because I was having so many coffees a day because you don’t sleep when you have a little one and in every postpartum intervention clinic I was having two sugars, so I slowly went down and I don’t have any sugar anymore and it’s been months (participant 5).

#### Theme 2. Patients Wanted More Regular Appointments

**Fig. 2:**
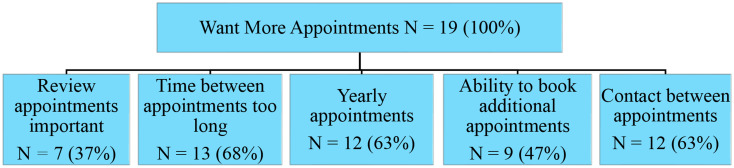
Patients wanted more appointments and identified five subthemes in this area

The second theme relating to the aim of exploring experiences was that 100% of participants reported wanting more appointments (*N* = 19; Fig. 2) and said they felt it important to have routine scheduled follow-up instead of a single appointment (*N* = 7, 37%): “The clinic is not like other clinic appointments with doctors where it’s like ‘here is what you have to do, off you go’ and there is no follow up and nobody cares how you are going. You truly care” (participant 16).

More than half of the participants (*N* = 12, 63%) described wanting annual appointments:

You need to see people every 12 months, so if people are slipping in their eating habits or starting to get high blood pressure, you might be able to get them more education to stop it. If you don’t see them, they might get worse, and nobody wants to have a heart attack when you have kids (participant 9).

Most participants (*N* = 13, 68%) reported that the time between the second appointment and third appointment was too long: “you should see people sooner because it is a preventative clinic and that 18 months to 5 years is a long time” (participant 17).

Participants (*N* = 9, 47%) also described wanting the flexibility to book additional appointments:

Appointments could be flexible based on whether we are still following the advice and how we are feeling. If someone is doing well and they don’t have any questions, then you could phone us and be like okay good job, keep going, any questions and we will see you in a while. But if someone is struggling, they could come in and be reviewed sooner (participant 11).

Most participants (*N* = 12, 63%) also talked about wanting additional contact between review appointments for additional support and mentioned that this could be done through closed online groups such as: “Facebook groups where we can connect to people in the same situation and support each other” (participant 1).

Some participants (*N* = 8, 42%) mentioned that phone calls (either scheduled or unscheduled) from the nurse practitioner to check in would support them to continue to follow recommendations, saying “even a quick phone call would keep you accountable” (participant 8). Some (*N* = 6, 32%) also described that email newsletters with general lifestyle recommendations would be beneficial to remind them to follow the advice and stay engaged: “newsletters would be good to remind us and with tips for everyone. For example, ‘try going for a 30 minute walk this week’ (participant 14)”.

### Aim 2. Patient Reported Barriers

#### Theme 3. Unclear Clinic Referral and Appointment Process

**Fig. 3:**
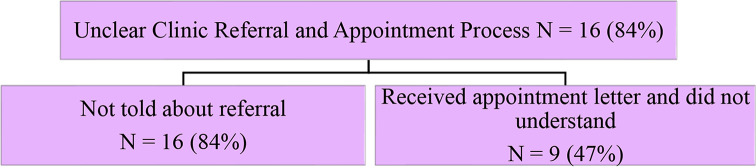
Patients felt there was an unclear clinic referral and appointment process, and identified two subthemes

This study also investigated reported barriers to clinic engagement. The first emerging theme relating to this aim was that the referral process to the clinic was unclear (Fig. 3). Most participants (*N* = 16, 84%) mentioned that nobody in their treating health care team had talked to them about a referral to the postpartum intervention clinic either during their antenatal care, hospital admission or at postnatal hospital discharge: “Nobody said anything about this clinic. If they said they were going to refer me, I wouldn’t have been so shocked when I got the letter in the mail” (participant 4). Almost half (*N* = 9, 47%) reported receiving an appointment letter and not understanding what it was for: “I had no clue. I didn’t know what the letter was. I almost put it in the bin and would have not come to the clinic” (participant 15).

#### Theme 4. Missing and Incorrect Appointment Letters and Forms

**Fig. 4:**
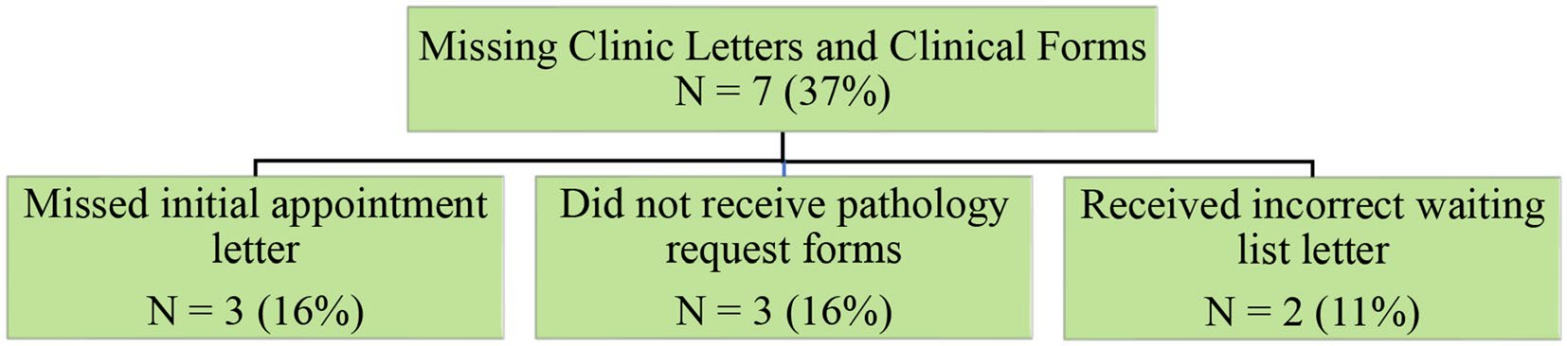
Patients reported missing and incorrect appointment letters and forms, and identified three subthemes

Another emerging theme that related to barriers was significant administrative errors including missing letters and pathology forms for the clinic (Fig. 4). Some participants (*N* = 3, 16%) described not receiving the initial appointment letter with pathology forms:

I didn’t receive appointment letters. I got this letter reminder one a couple of weeks before, but I didn’t get any blood forms, I had to call to them to send them out again, and then they didn’t come in time before my appointment, so I had to get them done after (participant 18).

Others (*N* = 3, 16%) described not receiving pathology forms: “I got a text message the day before and it said remember to get your bloods done but I didn’t know we had to and didn’t get any forms” (participant 13).

Additionally, two others (11%) mentioned receiving an incorrect waiting list letter instead of an initial appointment letter:

I got this weird letter that said I was on a waitlist, and I was confused because I didn’t know what it was about. I called the number on the waitlist letter and only then made an appointment. Otherwise, I would have been referred but never had an appointment (participant 16).

## Discussion

This was the first study to investigate the experiences of patients who attended a postpartum cardiovascular disease prevention clinic in Australia after having a pregnancy complication. The aims were to explore the experiences and barriers of patients who attended the clinic. Participants reported finding the clinic educational and wanted more appointments. Barriers to engagement reported by those who attended included an unclear referral and appointment process and missing letters and forms.

### Aim 1. Patient Reported Experiences

The first aim explored the experiences of the patients that have attended the postpartum intervention clinic. There were two main themes.

#### Theme 1. Patients Found the Postpartum Intervention Clinic Useful

Participants found the clinic useful, specifically describing it as informative and thorough in assessments. Patients reported that the lack of comprehensive information given in the clinic was a stark contrast to the little information they received after being diagnosed with a complication of pregnancy. This was consistent with another recent focus group study of antenatal experiences of preeclampsia or/and gestational diabetes that also reported receiving no or low information during antenatal care (Sandsæter et al., 2019). Improving the education regarding increased cardiovascular disease risk after complications of pregnancy could improve attendance and engagement in the clinic. For instance, studies engaging cardiac rehabilitation patients have found that individuals with greater health literacy (knowledge, understanding and application of health information) have higher appointment attendance and program completion than those with low health literacy (Aaby et al., 2017; Knudsen et al., 2020). This highlights the need for a standardized education process on the risk for these patients at postnatal discharge or postpartum review.

#### Theme 2. Patients Wanted More Regular Appointments

The second theme was that participants wanted more regular appointments, expressing that the gap between the second and third appointments was too long. Patients explained that 12-monthly appointments are needed in addition to the ability to book additional appointments and create opportunities for support and contact between appointments. The significance of these results was difficult to quantify considering the paucity of literature, but they are an important first step in understanding the best way to support this cohort of high-risk women. Participants described wanting social media groups to connect with people with the same lived experience, clinic educational newsletters and phone call check-ins from the nurse practitioner to further support them. Previous evidence supports this feedback from a similar, yet higher socioeconomic group of women with a history of preeclampsia in New South Wales, of whom 70% reported wanting information through web-based systems (Hutchesson et al., 2018). The effectiveness of online interventions at reducing modifiable cardiovascular disease risk factors in this cohort of women with previous preeclampsia has been proposed and is currently being conducted (Taylor et al., 2019).

### Aim 2. Patient Reported Barriers

The second aim investigated barriers that prevent attendance in the postpartum intervention clinic. There were two main themes.

#### Theme 3. Unclear Clinic Referral and Appointment Process

The first theme relating to barriers was that the referral and appointment process was unclear, with participants reporting not being told about being referred and not understanding the initial appointment and information letters. Studies into cardiac rehabilitation attendance have found that a strong recommendation by the referring provider to attend an intervention program results in higher enrollment and attendance rates (Williamson et al., 2020). This indicates the need for standardized clinical recommendations and education on the increased risk of cardiovascular disease for patients who have had a pregnancy complication. There also needs to be some form of communication with the patients about the clinic and a referral being made prior to the woman receiving the initial appointment letter. Previous attempts have been made to have the referring obstetrician speak with the patients being referred and give them an information pamphlet about the clinic. However, the findings of this study make it clear that this still does not happen. Phone calls from a nurse practitioner explaining why they have been referred to the clinic and an appointment could be trialed for a small group of patients to see if this improves later attendance.

#### Theme 4. Missing and Incorrect Appointment Letters and Forms

The second theme relating to barriers were missing clinic appointment letters and forms, with patients reporting not receiving initial appointment letters, not receiving pathology request forms or receiving an incorrect waiting list letter instead of an initial appointment letter. These significant administrative issues are disappointing, yet unfortunately common for outpatient clinics within busy public hospital settings. Other focus group studies into reported barriers for cardiac rehabilitation outpatient clinics found that not receiving an appointment letter is associated with non-attendance (Supervía et al., 2017). Phone calls from the postpartum intervention clinic team to people referred to the clinic with the appointment date and time could be trialed.

The barriers differed from those reported from women attending the Maternal Health Clinic in Canada (Chan et al., 2021). This clinic sees patients at six months postpartum after experiencing a pregnancy complication to assess, educate and reduce cardiovascular disease risk (Chan et al., 2021). These patients reported barriers including being too busy with childcare, difficulties attending the appointment date/time and deciding to access care with a general practitioner instead (Chan et al., 2021). The difference in reported barriers may have been because this study included both individuals who attended or did not attend the clinic (Chan et al., 2021). The reported barriers identified in this study are clinically significant and provide a clear target for improving engagement and attendance. However, any further investigation into perspectives and barriers needs to include people who attended their appointments and those who did not.

This study was the first to explore patient’s experiences of attending a postpartum outpatient cardiovascular prevention clinic for patients with pregnancy complications within Australia; however, there were some limitations that should be considered in future studies. First, the focus group cohort was not entirely representative of the whole postpartum intervention clinic cohort. The focus group cohort was older (*p* < 0.05) and less likely to be unemployed compared to the whole cohort (*p* = 0.06). Previous studies have found that cardiovascular patients who attend voluntary focus groups tend to have a higher socioeconomic status than the general eligible cohort (Guo et al., 2017). The Lyell McEwin Hospital services a low socioeconomic area with some of the highest rates of chronic disease and social disadvantage in the country (Guo et al., 2017). Therefore, the sample is not entirely reflective of the typical group referred and seen in the postpartum intervention clinic, and future endeavors to target a more varied cohort should be pursued.

This study also excluded non-English speaking patients due to practicality issues with conducting focus groups with interpreters. Approximately one-fourth of all patients referred to the clinic are non-English speaking, so it is likely that our data was not representative of the whole cohort (Aldridge et al., 2022). Further studies should include non-English speaking patients by conducting semi-structured one-on-one interviews with an interpreter, as this may identify improvements and barriers that have not previously been reported.

In addition, despite contacting all eligible clinic attendees for participation in this study, the uptake was relatively low with only nineteen clinic patients participating. We attempted to make focus group attendance as convenient as possible for participants by offering multiple dates and times on different days of the week across a two-week period and providing an after-hours option. Encouraging children to be brought to the sessions, offering gift vouchers to reimburse participants for their time and sending text message reminders the day prior were all strategies used to maximize the number of attendees. Further focus group studies should have greater flexibility in days and times, including weekends and more time after hours. Additionally, more rural patients may be included by using telehealth platforms. However, despite the small sample size we reached thematic saturation after the third focus group (Hennink et al., 2019). Other options to gather perspectives, including using questionnaires and telephone interviews, should also be considered in future. These options are less time intensive for participants and may have a greater participation rate.

Another limitation was that our sample did not include any participants with a history of placental abruption. This was likely due to the scarcity of this condition, as it only affects 2% of the total postpartum intervention clinic cohort and 1% of pregnancies in Australia (Farquhar et al., 2017). Further studies need to ensure that patients with a history of different types of pregnancy complications are included as they may have had different experiences that could further improve the clinic.

Further refinement of the postpartum intervention clinic model is needed to maximize consumer engagement and education, support patients in reducing their cardiovascular risk and minimize barriers to attending the service. One strategy would be to revise the appointment schedule to every 12 months and include the option to schedule additional appointments if needed. To improve the referral and appointment process, phone calls to patients explaining the link between pregnancy complications and cardiovascular disease, their referral to the clinic and their first appointment date and time should be trialed. Further studies with focus groups and questionnaires will need to be conducted with a larger sample to get further feedback from patients with more varied social and health backgrounds.

This study was the first to consider the perspectives of patients attending the only existing outpatient postpartum cardiovascular prevention clinic for people who have had a pregnancy complication within Australia. Improvements in the form of regular appointments, a clearer referral and appointment process, and improved correspondence will improve the clinic. The model can be replicated in other healthcare settings to become part of standardized care in the postpartum period for patients who have had a pregnancy complication. This targeted preventative clinic will educate patients, support a reduction in the risk of cardiovascular disease and improve their overall cardiac health.

## Data Availability

The datasets have not been made publicly available as the authors are not permitted to share datasets due to ethical requirements. Requests to access should be directed to Tegan Manthorpe, tegan.manthorpe@sa.gov.au.
