# Correction: Patient Experiences of a Postpartum Cardiovascular Disease Intervention Clinic for Pregnancy Complications

**DOI:** 10.1007/s10995-025-04083-w

**Published:** 2025-03-12

**Authors:** Tegan Manthorpe, Margaret Arstall, Prabha H. Andraweera, Emily Aldridge

**Affiliations:** 1 Adelaide Medical School, Robinson Research Institute, The University of Adelaide, Adelaide, South Australia Australia; 2 Department of Cardiology, Lyell McEwin Hospital, Adelaide, South Australia Australia

**Correction to: Maternal and Child Health Journal**

10.1007/s10995-025-04047-0

The original version of this article contained an error in Fig. 3. In this figure, the line numbers are present inside the image and published incorrectly.

For completeness and transparency, both the incorrect and correct versions are displayed below.

**Incorrect version**:


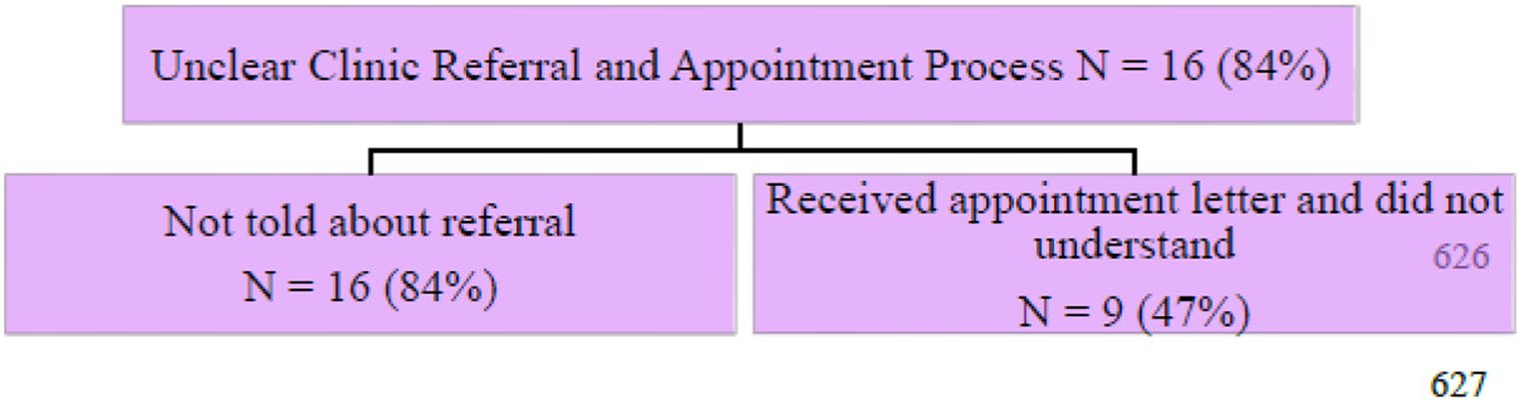


**Corrected version**:

**Fig. 3:**
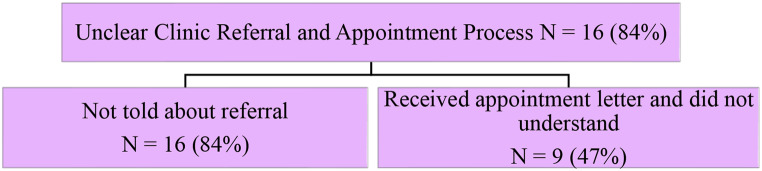
Patients felt there was an unclear clinic referral and appointment process, and identified two subthemes

The original article has been corrected.

